# Dietary curcumin encapsulated in nanostructured lipid carriers improves growth performance, feed utilization efficiency, and resistance to <em>Streptococcus agalactiae</em> in Nile tilapia (<em>Oreochromis niloticus</em>)

**DOI:** 10.14202/vetworld.2026.3156-3175

**Published:** 2026-07-20

**Authors:** Warut Kengkittipat, Manoj Tukaram Kamble, Sirikorn Kitiyodom, Jakarwan Yostawonkul, Gotchagorn Sawatphakdee, Kim D. Thompson, Seema Vijay Medhe, Saharuetai Jeamsripong, Nopadon Pirarat

**Affiliations:** 1 International Graduate Course of Veterinary Science and Technology (VST), Faculty of Veterinary Science, Chulalongkorn University, Bangkok 10330, Thailand.; 2 Center of Excellence in Wildlife, Exotic, and Aquatic Animal Pathology, Faculty of Veterinary Science, Chulalongkorn University, Bangkok 10330, Thailand.; 3 Department of Agricultural and Fishery Science, Faculty of Science and Technology, Prince of Songkla University, Pattani 94000, Thailand.; 4 National Nanotechnology Center (NANOTEC), National Science and Technology Development Agency (NSTDA), Pathumthani 12120, Thailand.; 5 ASEAN Institute for Health Development, Mahidol University, Salaya, Putthamonthon 73170, Nakhon Pathom, Thailand.; 6 Research Unit in Microbial Food Safety and Antimicrobial Resistance, Department of Veterinary Public Health, Faculty of Veterinary Science, Chulalongkorn University, Bangkok 10330, Thailand.

**Keywords:** aquaculture, curcumin, disease resistance, feed efficiency, nanostructured lipid carriers, Nile tilapia, streptococcosis, sustained release

## Abstract

**Background and Aim:**

Streptococcosis caused by *Streptococcus agalactiae* represents a major constraint to Nile tilapia (*Oreochromis niloticus*) aquaculture, resulting in substantial economic losses. Curcumin (CUR) possesses antioxidant, antimicrobial, and immunomodulatory properties, but its practical application is limited by poor aqueous solubility and low bioavailability. This study aimed to develop CUR encapsulated in nanostructured lipid carriers (CUR-NLCs) and evaluate their effects on growth performance, feed utilization efficiency, and resistance against *S. agalactiae* infection in Nile tilapia.

**Materials and Methods:**

CUR-NLCs were synthesized using a phase-inversion compositional technique and characterized for particle size, polydispersity index, zeta potential, encapsulation efficiency, and release behavior. A 60-day feeding trial was conducted using four dietary treatments: a control diet, a free CUR diet (2000 mg/kg feed), CUR-NLCs containing an equivalent amount of CUR, and blank nanostructured lipid carriers. Growth performance, feed utilization indices, hepatosomatic index, and length–weight relationships were evaluated. Antibacterial activity against *S. agalactiae* isolates was determined, followed by experimental challenge with strain ENC06 to assess survival responses.

**Results:**

CUR-NLCs exhibited favorable physicochemical characteristics, including a mean particle size of 215.6 nm, a low polydispersity index (0.145), and a high encapsulation efficiency (98.79%), along with sustained-release properties. CUR-NLCs produced larger inhibition zones against *S. agalactiae* than free CUR. Fish receiving the CUR-NLC diet demonstrated significantly higher final body weight, weight gain, specific growth rate, feed intake, and protein efficiency ratio than the control group (p < 0.05). Positive allometric growth (b = 3.278) and improved relative condition factor were observed in CUR-NLC-fed fish. Following bacterial challenge, CUR-NLC supplementation resulted in the lowest mortality (11.1%), highest survival (88.9%), and highest relative percent survival (82.4%). Cox proportional hazards analysis indicated an approximately 86% reduction in mortality risk compared with infected controls.

**Conclusion:**

Dietary administration of CUR encapsulated in nanostructured lipid carriers improved growth performance, feed utilization efficiency, and post-challenge survival of Nile tilapia. These findings demonstrate that CUR-NLCs represent a promising phytobiotic-based nutritional strategy for enhancing fish health and promoting sustainable tilapia aquaculture.

## INTRODUCTION

Aquaculture has experienced substantial global growth over the past several decades and, in 2022, accounted for just over half of total aquatic animal production worldwide, marking the first time farmed production exceeded that of capture fisheries [1]. Projections indicate that global aquaculture production will increase to approximately 111 million tons by 2032, underscoring its growing importance in meeting the rising global demand for high-quality protein [1, 2]. Within freshwater aquaculture systems, *Oreochromis niloticus *(Nile tilapia) remains one of the most extensively farmed species, ranking second globally with an estimated production of 5.3 million tons in 2022 [3]. However, the intensification of aquaculture systems, although enhancing productivity, has simultaneously increased vulnerability to infectious disease outbreaks, resulting in considerable economic losses and threatening production sustainability [4, 5].

Streptococcosis is recognized as one of the most economically devastating bacterial diseases affecting tilapia farming worldwide [6, 7]. *Streptococcus agalactiae* (Group B Streptococcus) is responsible for recurrent disease outbreaks characterized by septicemia and meningoencephalitis, leading to high mortality rates and reduced profitability of aquaculture operations [8, 9]. Streptococcosis caused by *S. agalactiae* represents a major economic burden in tilapia aquaculture, with global losses estimated to exceed USD 1 billion annually [10]. Under intensive production conditions, outbreaks frequently result in mortality rates ranging from 30% to 70%, substantially compromising production efficiency, farm profitability, and trade sustainability in major tilapia-producing regions [11, 12]. Although disease management strategies, including vaccination, selective breeding for resistance, and probiotic supplementation, have shown promise, their implementation remains inconsistent, and the level of protection achieved is often incomplete under commercial farming conditions [11, 13, 14]. Consequently, the development of complementary and sustainable approaches to improve disease resilience remains a major research priority.

Traditionally, bacterial diseases in tilapia farming have been controlled primarily through antimicrobial drugs and chemical treatments. However, the prolonged and widespread use of these agents has resulted in the emergence and dissemination of antimicrobial resistance, reduced therapeutic efficacy, and increasing concerns regarding environmental contamination and food safety [15–17]. These interconnected challenges have created an urgent need to shift toward more sustainable and biologically compatible disease management strategies. Consequently, increasing attention has been paid to alternative approaches that enhance fish health and disease resistance without exacerbating ecological risks [18, 19]. In this context, phytobiotics, probiotics, prebiotics, and synbiotics have emerged as promising options because they provide antimicrobial and immunomodulatory benefits while avoiding many of the limitations associated with conventional antibiotic therapies [16, 20–23].

Among phytobiotics, Curcumin (CUR), a naturally occurring polyphenol derived from *Curcuma longa*, has attracted considerable scientific interest because of its potent antioxidant and anti-inflammatory properties [24, 25]. Beyond these activities, CUR exhibits hepatoprotective effects and modulates immune responses, thereby contributing to improved physiological condition and enhanced stress tolerance in aquatic organisms [26–30]. In addition, its antibacterial activity against several fish pathogens further underscores its potential for disease management [26, 27, 31]. Despite these functional advantages, the practical use of CUR remains limited by poor aqueous solubility, chemical instability, and low bioavailability, which collectively reduce its efficacy when incorporated into conventional feed systems [32–34].

Nanostructured lipid carriers (NLCs) have emerged as efficient delivery systems for hydrophobic and chemically unstable bioactive compounds, such as CUR, because they enhance molecular stability, facilitating intestinal absorption and enabling controlled release [35, 36]. Compared with conventional solid lipid nanoparticles, NLCs possess a less ordered lipid matrix formed by combining solid and liquid lipids, thereby enhancing drug-loading capacity and minimizing compound expulsion during storage [37]. In contrast to liposomal systems, NLCs offer superior physical stability, greater compatibility with lipophilic compounds, and enhanced protection of encapsulated phytochemicals under gastrointestinal conditions [38]. By incorporating CUR into a lipid-based matrix, NLCs may facilitate sustained physiological exposure and prolonged biological activity, thereby enabling compound-specific effects extending beyond growth promotion [39]. Previous studies by our group and others have demonstrated the utility of lipid-based nanocarriers for delivering various bioactive compounds in aquatic species, including hormonal, anesthetic, and phytogenic substances [30, 40–43]. Nevertheless, these studies involved compounds with physicochemical characteristics and biological activities fundamentally different from those of CUR, and the biological implications of CUR delivery through NLCs in Nile tilapia remain largely unexplored. Compared with other conventional nano-CUR delivery systems, NLCs may provide superior physicochemical stability and sustained-release characteristics suitable for long-term dietary application of hydrophobic phytochemicals.

Although the beneficial effects of CUR on growth performance, antioxidant status, and immune responses have been reported in several aquatic species, its practical application in aquafeeds remains limited by poor solubility, instability, and low bioavailability. Nanoencapsulation approaches have been investigated to improve the delivery of various bioactive compounds; however, studies specifically evaluating CUR-loaded NLC systems in Nile tilapia remain scarce. Furthermore, previous investigations have mainly focused on physicochemical characterization or isolated biological responses, with limited attention given to the integrated effects of nanoencapsulated CUR on growth performance, feed utilization efficiency, hepatosomatic condition, growth allometry, antibacterial activity, and post-challenge survival against *S. agalactiae*. In addition, information regarding the ability of sustained-release CUR delivery systems to enhance disease resistance and production efficiency in Nile tilapia under experimental challenge conditions remains insufficient. Therefore, a comprehensive evaluation of CUR encapsulated in NLCs (CUR-NLCs) as a functional dietary strategy for improving both productive performance and host resilience is warranted.

Accordingly, the present study was undertaken to develop and comprehensively evaluate CUR-NLCs, with particular emphasis on their physicochemical characteristics and their effects on growth performance, feed utilization efficiency, hepatosomatic condition, length–weight relationships, and resistance to streptococcosis in Nile tilapia. In addition to *in vitro* antibacterial assessment, the study investigated post-challenge survival responses following experimental exposure to *S. agalactiae* ENC06, a serotype Ia strain frequently associated with streptococcosis outbreaks in tilapia farming systems in Thailand. By integrating physicochemical characterization with biological and disease resistance evaluations, this study aimed to clarify the potential of CUR delivered via NLCs as a sustainable, phytochemical-based nutritional strategy to enhance fish health, disease resilience, and production efficiency in Nile tilapia aquaculture.

## MATERIALS AND METHODS

### Ethical approval

All procedures involving experimental animals were reviewed and approved by the Chulalongkorn University Animal Care and Use Committee, Chulalongkorn University, Bangkok, Thailand (Approval No. 2431095). All experiments were conducted in accordance with institutional animal welfare guidelines and applicable regulatory standards. Appropriate measures were implemented throughout the study to minimize stress and discomfort to the fish. During challenge experiments, fish exhibiting severe loss of equilibrium, inability to feed, or unresponsiveness were considered moribund and were humanely euthanized in accordance with approved ethical guidelines.

### Study period and location

The study was conducted from May to August 2025 at the Center of Excellence in Wildlife, Exotic, and Aquatic Animal Pathology, Faculty of Veterinary Science, Chulalongkorn University, Bangkok, Thailand. Physicochemical characterization and biological evaluations of CUR-NLCs, including antibacterial assays, feeding trials, and challenge experiments, were carried out under controlled laboratory conditions. The feeding experiment was conducted over 60 days, followed by a 15-day post-challenge observation period.

### Study design

Prior to the feeding trial, Nile tilapia fingerlings (n = 600; initial body weight, 6-8 g) were treated prophylactically with 50 ppm formalin and acclimatized for 14 days in fiberglass tanks with a working volume of 150 L supplied with continuously aerated, flow-through dechlorinated tap water.

Following acclimatization, fish were randomly allocated to four dietary treatments comprising a basal control diet, free CUR, CUR-NLC, and blank NLC. Each treatment was performed in triplicate, with 50 fish stocked per tank.

Throughout the 60-day feeding period, environmental conditions were monitored daily to ensure stable culture conditions. Water temperature ranged from 26°C to 28°C, dissolved oxygen concentrations ranged from 5.34 to 5.78 mg/L, and pH values ranged from 7.38 to 8.20. Approximately 30%-50% of the water volume was exchanged daily to maintain suitable water quality. Fish were fed their respective diets twice daily at a feeding rate corresponding to 3% of total body weight.

Blinding was not performed during feed preparation, feeding, sampling, or challenge procedures because the dietary treatments were visibly distinguishable.

### Chemicals

CUR (95% purity, high-performance liquid chromatography grade; Batch No. NIS21070222-TMR-01) was obtained from Primo Trading Co., Ltd. (Bangkok, Thailand). Lipid excipients, including sorbitan oleate, cetearyl alcohol, cocoglucoside, polyoxyethylene (20) sorbitan monolaurate (Tween 20), poloxamer 188, and glycerol, were purchased from Croda (Thailand) Co., Ltd. (Bangkok, Thailand). Ethoxydiglycol was supplied by Myskin Recipe Co., Ltd. (Bangkok, Thailand). All other chemicals and reagents used in this study were of analytical grade and suitable for experimental and biological applications.

### Formulation of CUR-NLCs

CUR-NLCs were prepared using a phase-inversion compositional approach coupled with high-energy homogenization. This formulation strategy was selected to facilitate stable incorporation and sustained dietary delivery of CUR, whose low aqueous solubility and chemical instability limit its effectiveness in conventional feed formulations. The preparation protocol followed a previously validated NLC platform [40], and formulation parameters were maintained to enable direct evaluation of compound-specific biological effects rather than differences arising from carrier architecture.

Briefly, 200 mg of CUR was incorporated into a lipid phase comprising a solid lipid component (cetearyl alcohol-cocoglucoside blend, 1.0 g), a liquid lipid component (sorbitan oleate, 3.0 g), and ethoxydiglycol (3.0 g), and the mixture was continuously agitated at 60°C to ensure complete solubilization. Accordingly, the formulation contained an approximate solid-to-liquid lipid ratio of 1:3. Separately, an aqueous phase containing polyoxyethylene (20) sorbitan monolaurate (3.0 g), poloxamer 188 (2.0 g), and glycerol (2.5 g) was prepared in deionized water and maintained at the same temperature. The heated aqueous phase was gradually added to the lipid mixture and homogenized at 500 rpm for 5 min to obtain a preliminary dispersion. Subsequently, ultrasonic processing was performed at 30% power output for 5 min using intermittent 30-s cycles with resting intervals to avoid excessive heat generation. Following sonication, the CUR-NLC formulation was allowed to equilibrate to ambient temperature before further physicochemical and biological characterization.

### Physicochemical characterization of CUR-NLCs

The physicochemical characteristics of CUR-NLCs, including particle size, size distribution, and surface charge, were determined using dynamic light scattering with a Zetasizer Nano ZS system (Malvern Instruments, Malvern, UK). Samples were diluted 50-fold with deionized water before analysis to minimize light-scattering interference. All measurements were performed at 25°C, and each parameter was measured in triplicate.

Nanoparticle morphology was examined using transmission electron microscopy (HT7800; Hitachi High-Tech Corporation, Tokyo, Japan) operated at 80 kV [44]. For sample preparation, CUR-NLC dispersions were diluted (1:50) with deionized water adjusted to pH 7.0 and deposited onto carbon-coated copper grids. Excess liquid was removed using filter paper, and the grids were stained with uranyl acetate staining solution for 2 min, rinsed with deionized water, air-dried, and subsequently examined microscopically. Transmission electron microscopy analysis was used for qualitative morphological assessment.

### Encapsulation capacity and release characteristics of CUR-NLCs

The proportion of CUR successfully incorporated into NLCs was evaluated and compared with free CUR preparations using centrifugal separation based on molecular size exclusion. Regenerated cellulose ultrafiltration devices with a nominal cutoff of 30 kDa (Amicon Ultra-15, Merck Millipore Ltd., Burlington, MA, USA) were used in accordance with a validated analytical procedure [40]. For each formulation, 1.5 mL was transferred to filtration units and centrifuged according to the manufacturer's instructions, thereby separating carrier-associated CUR from the freely dissolved fraction. The filtrate containing unassociated CUR was recovered by solvent extraction as previously described by [41], passed through a 0.2 μm nylon membrane, and quantified by high-performance liquid chromatography.

Quantitative determination of CUR was performed using a Waters liquid chromatography system equipped with a photodiode array detector (Model 2998; Waters Corporation, Milford, MA, USA). Separation was achieved on a reversed-phase C18 column (Ascentis, 5 μm, 250 × 4.6 mm; Sigma-Aldrich, St. Louis, MO, USA). The mobile phase consisted of acetonitrile and methanol (60:40, v/v) delivered at a flow rate of 1.0 mL/min. The sample volume was 20 μL, and detection was performed at 240 nm. Each chromatographic run was completed within 15 min.

Encapsulation efficiency (EE) was calculated using the following equation:


\[
EE(\%)=\frac{\left({C}_{i},{C}_{f}\right)}{{C}_{i}}\times 100
\]

where \({C}_{i}\)represents the initial CUR concentration and \({C}_{f}\)corresponds to the amount of CUR detected in the filtrate.

The loading capacity (LC), which describes the proportion of CUR entrapped relative to the total lipid mass, was calculated as follows:


\[
LC(\%)=\frac{\left({C}_{i},{C}_{f}\right)}{Total lipid mass}\times 100
\]

The release profile of CUR from CUR-NLCs was investigated under simulated physiological conditions using a diffusion-controlled dialysis technique. Briefly, 1.5 mL of CUR-NLC dispersion was transferred into dialysis membranes (molecular weight cutoff 3.5 kDa; Merck Millipore) and immersed in 30 mL of phosphate-buffered saline (pH 6.8) containing ethanol (95:5, v/v) to maintain sink conditions [40]. The system was incubated at 28°C in an orbital shaking incubator (Vision Scientific Co., Daejeon, South Korea) operating at 200 rpm.

At predetermined intervals (0-48 h), aliquots were withdrawn from the release medium and replaced with fresh buffer to maintain a constant volume. The released CUR concentration at each time point was determined using high-performance liquid chromatography. Release kinetics were analyzed using the Avrami model:


\[
R=1-exp⁡[-(kt{)}^{n}]
\]

where \(R\)denotes cumulative release at time \(t\), \(k\)is the apparent release rate constant, and \(n\)represents the release mechanism.

### Functional group analysis

To investigate molecular interactions between CUR and the lipid matrix, Fourier-transform infrared spectroscopy was performed [45]. Spectra of free CUR and CUR-NLCs were obtained using a Nicolet Summit Pro Fourier-transform infrared spectrometer (Thermo Scientific, Waltham, MA, USA). Spectra were recorded over the range of 500-4000 cm⁻¹ at a resolution of 4 cm⁻¹.

Samples were prepared by mixing formulations with potassium bromide at a 1:100 ratio, followed by grinding and pellet formation. Each spectrum was generated from 64 accumulated scans, and all analyses were performed in triplicate to ensure reproducibility.

### Evaluation of antibacterial activity

The antibacterial activity of free CUR and CUR-NLCs was evaluated against three *S. agalactiae* isolates (FNA07, FPrA02, and ENC06) obtained from diseased Nile tilapia in Thailand. The assay was performed using the agar diffusion method. All analyses were conducted in triplicate for each isolate and formulation.

Bacterial isolates were cultured on tryptic soy agar supplemented with 5% sheep blood and incubated at 28°C for 24 h [46]. Fresh colonies were suspended in sterile 0.85% saline and adjusted to a turbidity equivalent to a 0.5 McFarland standard. Bacterial suspensions were evenly spread onto Mueller-Hinton agar supplemented with 5% sheep blood. Wells measuring 6 mm in diameter were prepared aseptically, and 50 μL of either free CUR or CUR-NLC formulation was added to each well. Plates were incubated at 28°C for an additional 24 h, and antibacterial activity was determined by measuring the diameters of growth inhibition zones (IZ) with a calibrated Vernier caliper to a precision of ±0.02 mm.

**Determination of minimum inhibitory concentration (MIC) and minimum bactericidal concentration (MBC)**

The inhibitory and bactericidal activities of CUR and CUR-NLCs were evaluated using the broth dilution method [44]. Stock formulations were serially diluted twofold in Mueller-Hinton broth to obtain concentrations ranging from 2,000 to 3.9 ppm. Aliquots were transferred into sterile 96-well microplates, and each well received 100 μL of bacterial suspension adjusted to a final concentration of 1 × 10⁶ colony-forming units/mL.

Following inoculation, plates were incubated aerobically at 28°C for 24 h. The MIC was defined as the lowest concentration that completely inhibited visible bacterial growth. To determine the MBC, aliquots from wells without visible turbidity were plated onto Mueller-Hinton agar and incubated under identical conditions. The MBC was defined as the lowest concentration at which no bacterial colonies were recovered.

### Preparation of experimental feed

Commercial tilapia pellets (CP-7710) were used as the basal diet (moisture, 7.6%; crude protein, 30.5%; crude lipid, 6.5%; and ash, 8.2%, according to the manufacturer's specifications) and were externally coated with CUR, CUR-NLCs, or blank NLC formulations. Because the coating formulations were applied at relatively low inclusion levels relative to the total feed mass, substantial alteration of the proximate nutritional composition of the commercial basal diet was not expected.

Coating solutions were prepared at a final concentration of 2 mg/mL and applied at a rate of 1 mL/g feed [45], resulting in an approximate CUR inclusion level of 2000 mg/kg feed. The final dietary CUR concentration was calculated from the formulation concentration and the coating volume applied per unit of feed. Analytical verification of CUR retention in coated pellets after drying and storage was not performed in the present study. However, the coating procedure was standardized across all experimental diets to ensure consistency among treatments. This supplementation level was selected based on previously reported dietary CUR inclusion levels used in Nile tilapia and other fish species [27, 47].

The formulated NLC dispersions were homogeneous, low-viscosity fluids at ambient temperature, facilitating uniform distribution over the commercial feed pellets. Pellets were gently agitated using a sterile stainless-steel spatula to ensure uniform surface coating. Control diets were prepared using distilled water in place of active formulations. Following coating, the feeds were dried overnight at room temperature(28–30°C) and subsequently stored at 4°C until use.

During the feeding period, fish readily accepted all experimental diets, including pellets coated with CUR and CUR-NLC formulations, without observable feed rejection or abnormal feeding behavior. In addition, no visible discoloration of the rearing water or apparent residue associated with CUR or CUR-NLC leaching was observed during feeding, indicating acceptable coating adherence and physical stability of the supplemented pellets under the experimental conditions.

### Evaluation of growth performance and feed utilization

Fish growth performance and feed utilization efficiency were evaluated on days 30 and 60 of the feeding trial. The variables assessed included Weight gain (WG), Specific growth rate (SGR), mean daily intake (MDI), feed conversion ratio (FCR), protein efficiency ratio (PER), and hepatosomatic index (HSI). These parameters were calculated using established methods in aquaculture nutrition studies [48].

WG (g/fish) was calculated as follows:


\[
WG=FW-IW
\]

where FW and IW represent final and initial body weight, respectively.

SGR (%/day) was calculated as:


\[
SGR=100\times \frac{ln⁡FW-ln⁡IW}{T}
\]

where T denotes the duration of the feeding period.

MDI (g/fish/day) was determined as:


\[
Mean daily intake=\frac{Total feed intake/T}{Number of fish per tank}
\]

FCR was calculated as:


\[
FCR=\frac{Feed intake}{Wet weight gain}
\]

PER was calculated using the following equation:


\[
PER=\frac{Wet weight gain}{Protein intake}
\]

HSI (%) was calculated as:


\[
HSI=\frac{Liver weight}{Body weight}\times 100
\]

Length–weight relationship, growth pattern, and relative condition factor (Kn)

At the end of the 60-day feeding period, body size scaling and physiological condition were evaluated. The relationship between body weight and total length was determined using the allometric equation described by Pauly [49]:


\[
W=a{L}^{b}
\]

where W denotes body weight (g), L denotes total length (cm), and a and b are the intercept and slope of the regression model, respectively.

To estimate these parameters, the equation was transformed into logarithmic form:


\[
log⁡W=log⁡a+blog⁡L
\]

Growth patterns were interpreted according to the value of b. A value of b = 3 indicated isometric growth, whereas values greater than or less than 3 represented positive and negative allometric growth, respectively [50].

The relative condition factor (Kn) was further evaluated according to the method described by Le Cren [51]:


\[
Kn=\frac{{W}_{o}}{{W}_{c}}
\]

where \({W}_{o}\)is the observed body weight and \({W}_{c}\) is the predicted body weight estimated from the population-derived length-weight equation \(\left(W,a{L}^{b}\right)\). The predictive equation was generated using pooled measurements from all fish sampled on day 60. Values of Kn >1 indicated relatively better physiological condition, whereas values <1 indicated poorer condition.

### <H2>Disease challenge and survival analysis

For the challenge experiment, the *S. agalactiae* isolate ENC06 was selected because it exhibited greater susceptibility to CUR-NLC treatment during antibacterial screening and has been repeatedly associated with severe streptococcosis outbreaks in Nile tilapia farms in Thailand [52, 53].

At the end of the feeding trial, 30 fish from each dietary treatment were randomly selected for the challenge study. Before inoculation, fish were anesthetized with clove oil at a concentration of 20 mg/L to minimize handling stress and ensure humane treatment [54].

Experimental infection was performed by intraperitoneal injection of 100 μL bacterial suspension containing 1 × 10⁶ colony-forming units/mL of *S. agalactiae* ENC06. The challenge dose was selected based on a preliminary determination of the median lethal dose using the same strain under identical experimental conditions, to establish a sublethal dose appropriate for survival analysis.

Following inoculation, fish were transferred immediately to well-aerated recovery tanks, and handling time was minimized. Mortality was monitored daily during the 15-day post-challenge period. Confirmation of infection was achieved through re-isolation of *S. agalactiae* from kidney, spleen, and brain tissues of moribund and freshly dead fish.

During the challenge period, water temperature, pH, and dissolved oxygen remained within optimal ranges at 29.9 ± 0.05°C, 7.37 ± 0.06, and 4.97 ± 0.04 mg/L, respectively.

Host resistance was evaluated using cumulative mortality and relative percent survival (RPS) according to the method described by Amend [55]:


\[
\begin{aligned}
Cumulative mortality (\%)=\frac{Number of dead fish}{Initial number of fish}\times 100 \\
RPS(\%)=\left[1,\frac{Mortality percentage in treated group}{Mortality percentage in control group}\right]\times 100
\end{aligned}
\]

### Statistical analysis

Data were analyzed using SPSS Statistics version 29 (IBM Corp., Armonk, NY, USA). Before analysis, the normality of the residuals was assessed using the Shapiro-Wilk test, and the homogeneity of variance was evaluated using Levene's test.

The tank was considered the experimental unit, whereas measurements from individual fish were treated as subsamples and averaged before analysis. Parameters measured at days 30 and 60 were analyzed using repeated-measures analysis of variance, with time considered the within-subject factor and dietary treatment considered the between-subject factor.

Variables measured at a single time point were analyzed using one-way analysis of variance followed by Tukey's honestly significant difference post hoc test. Survival responses were analyzed using Kaplan-Meier survival curves, and group differences were evaluated using the log-rank test and Cox proportional hazards regression analysis.

Relationships among nanoparticle physicochemical characteristics, antibacterial activity, growth-related variables, and disease resistance parameters were investigated using Pearson's correlation analysis. Statistical significance was accepted at p < 0.05.

## RESULTS

### Physicochemical profile of CUR-loaded NLCs

Dynamic light scattering analysis showed that CUR incorporation increased the mean particle size of the NLC formulation. CUR-NLCs had a mean diameter of 215.6 ± 2.6 nm compared with 177.0 ± 3.2 nm for the unloaded NLC formulation (Table 1). CUR-NLCs also exhibited a more homogeneous size distribution, as indicated by a lower polydispersity index (0.145 ± 0.003) than blank NLCs (0.269 ± 0.034). Surface charge analysis showed moderately negative zeta potentials of −17.5 ± 0.78 mV for CUR-NLCs and −23.12 ± 0.9 mV for blank NLCs. CUR incorporation into the lipid matrix was highly efficient, with an encapsulation efficiency of 98.79 ± 1.51% and a loading capacity of 2.82% (w/w).

**Table 1:** Physicochemical characteristics of nanostructured lipid carrier and curcumin encapsulated in nanostructured lipid carrier formulations.

**Formulation**	**NLC**	**CUR-NLC**
Size (nm)	177.0 ± 3.2	215.6 ± 2.6
Polydispersity index	0.269 ± 0.034	0.145 ± 0.003
Zeta potential (mV)	−23.12 ± 0.9	−17.5 ± 0.78
Encapsulation efficiency (%)	ND	98.79 ± 1.51
Loading capacity (%)	ND	2.82 ± 0.04

Values are presented as mean ± standard error of triplicate samples. ND: not detected. NLC = Nanostructured lipid carrier, CUR-NLC = curcumin encapsulated in nanostructured lipid carriers.

**Figure 1:**
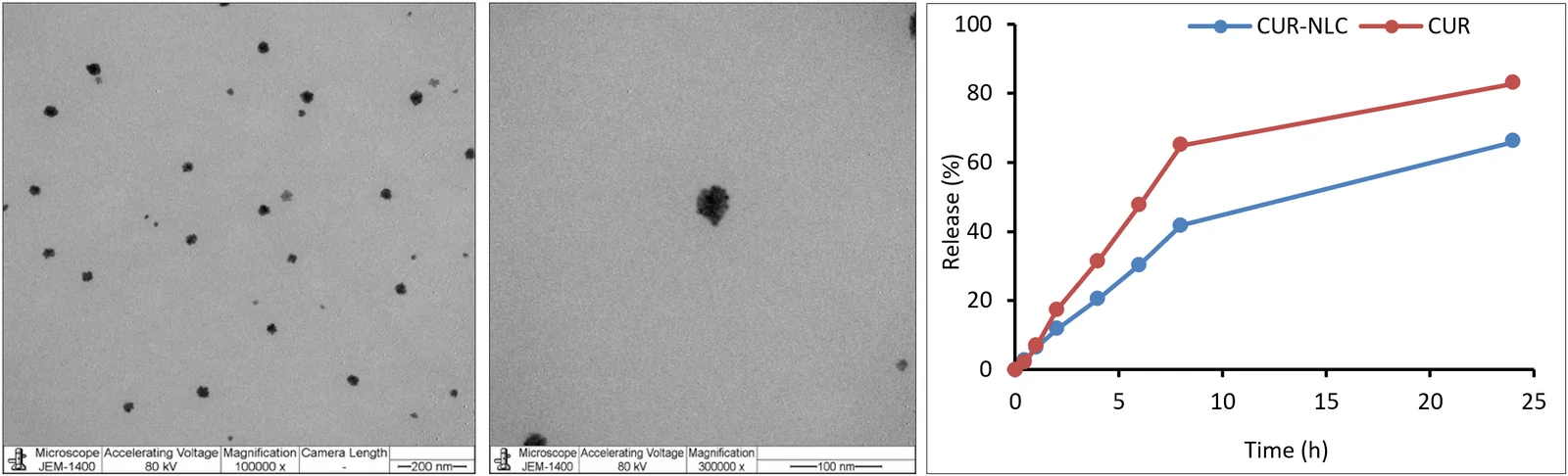
Morphology and release profile of curcumin encapsulated in nanostructured lipid carriers (CUR-NLCs). (A and B) Transmission electron microscopy images showing the spherical morphology and uniform distribution of CUR-NLCs at scale bars of 200 nm and 100 nm, respectively. (C) *In vitro* cumulative release profiles of free CUR and CUR-NLCs under simulated physiological conditions over 48 h. CUR-NLCs exhibited an initial release phase followed by sustained-release behavior, indicating efficient encapsulation and controlled-release characteristics. Data are presented as mean ± standard error (n = 3). Statistical comparisons between free CUR and CUR-NLC formulations at corresponding time points were performed using independent-samples t-tests. Significant differences in cumulative release were observed at 0.5 h (p < 0.05) and from 2 to 24 h (p < 0.001), whereas no significant differences were detected at 1 h and 48 h (p > 0.05).

Morphological assessment using transmission electron microscopy confirmed the formation of well-defined, spherical nanoparticles with a uniform appearance (Figures 1A and 1 B). The images indicated nanoscale particles in the expected size range of approximately 200 nm, consistent with dynamic light scattering measurements. These observations were interpreted qualitatively, considering possible effects of sample preparation.

Cumulative release profiles of CUR are shown in Figure 1C. CUR-NLCs exhibited an initial release phase followed by sustained release over 48 h, indicating prolonged release kinetics compared with free CUR, which showed a less sustained release pattern over the same period. Independent-samples t-test analysis demonstrated significant differences in cumulative release between free CUR and CUR-NLC formulations at multiple time points, particularly from 2 to 24 h (p < 0.05). The greatest differences were observed from 4 to 8 h (t = −16.678 to −21.768, p < 0.001), during which CUR-NLCs exhibited markedly greater cumulative release than free CUR. No statistically significant difference was detected at 48 h (p > 0.05).

### Functional group analysis

**Figure 2:**
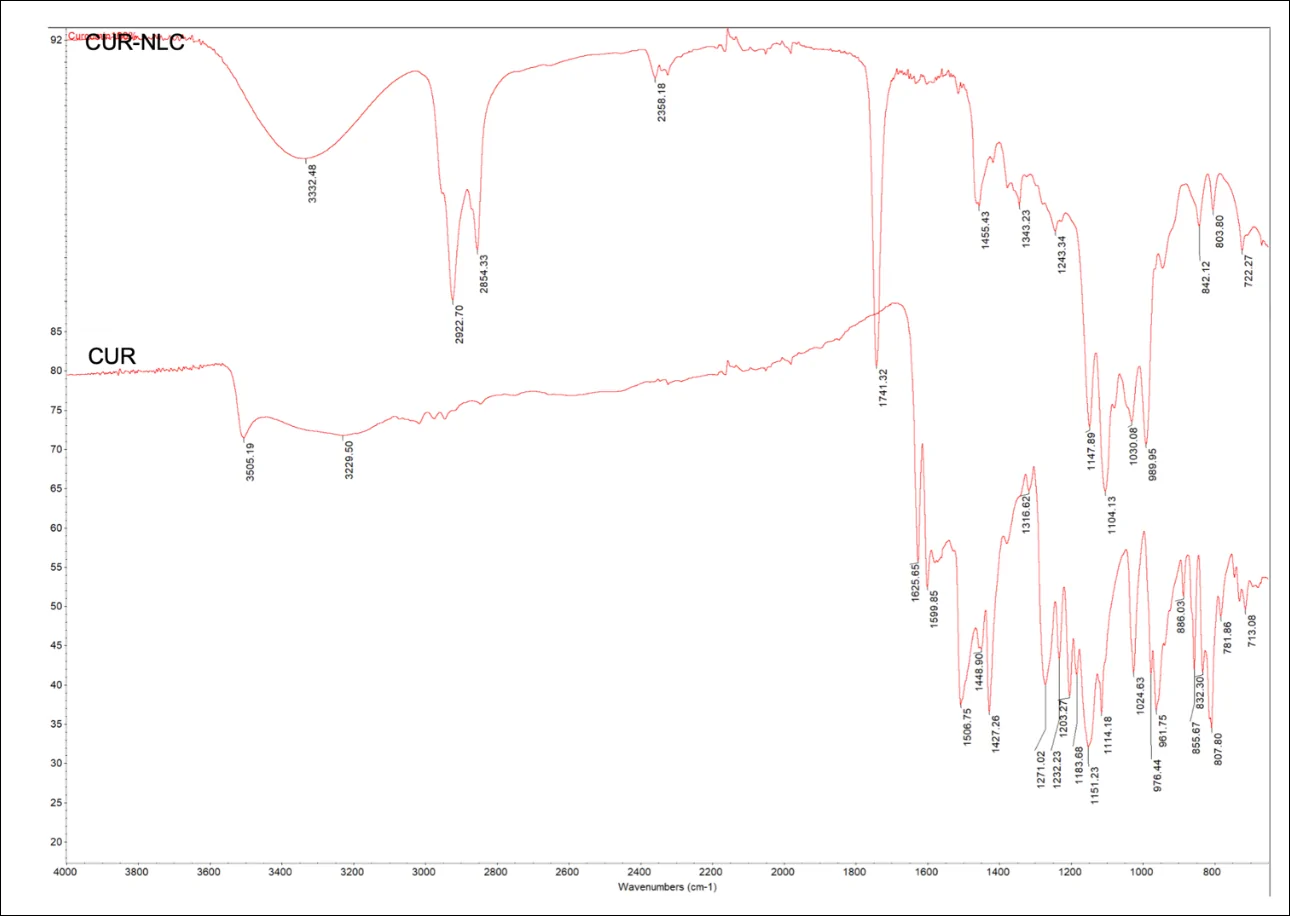
Fourier-transform infrared spectra of curcumin (CUR) and CUR encapsulated in nanostructured lipid carriers (CUR-NLCs). The bottom spectrum represents CUR, and the top spectrum represents CUR-loaded into NLCs. Characteristic shifts and intensity changes in functional group peaks support the interaction of CUR with the lipid matrix, indicating successful encapsulation within the CUR-NLC system.

Figure 2 illustrates the Fourier-transform infrared spectra of free CUR and CUR-NLCs, and the major absorption peaks and functional group assignments are summarized in Supplementary Table S1. In the CUR-NLC formulation, the characteristic O–H stretching vibration of CUR, observed near 3508 cm⁻¹, appeared shifted and showed lower band intensity. The C=O stretching vibration at approximately 1736 cm⁻¹ also shifted after incorporation into NLCs. Peaks associated with C=C stretching at approximately 1600–1625 cm⁻¹ and aromatic ring vibrations appeared broader and less intense in CUR-NLCs. Additional changes were observed in the 1300–1400 cm⁻¹ region, corresponding to C–O stretching and C–H bending vibrations.

### Antibacterial activity of CUR formulations

Table 2 summarizes the antibacterial activity of CUR formulations against *S. agalactiae* isolates. CUR-NLCs produced significantly larger IZ values (14–15 mm) than free CUR (6 mm) across all tested isolates (p < 0.05). MIC values were 1000 ppm for both CUR and CUR-NLCs across all isolates, whereas MBC values were 2000 ppm for both formulations.

**Table 2:** Antibacterial response of free curcumin (CUR) and CUR encapsulated in nanostructured lipid carriers (CUR-NLCs) against Streptococcus agalactiae isolates FPrA02, FNA07, and ENC06.

**Parameters**	**Isolate**	**CUR**	**CUR-NLC**
IZ (mm)	FPrA02	6 ± 0ᵃ	15 ± 1ᵇ
	FNA07	6 ± 0ᵃ	14 ± 2ᵇ
	ENC06	6 ± 0ᵃ	15 ± 2ᵇ
MIC (ppm)	FPrA02	1000	1000
	FNA07	1000	1000
	ENC06	1000	1000
MBC (ppm)	FPrA02	2000	2000
	FNA07	2000	2000
	ENC06	2000	2000

Data are presented as mean values with corresponding standard error calculated from three independent replicates. Different lowercase superscript letters within the same row indicate statistically significant differences among treatments.

### Growth performance and feed utilization

Growth performance and feed utilization values measured at 30 and 60 days are summarized in Table 3. At day 30, fish fed the CUR-NLC-supplemented diet showed significantly higher FW and WG than the control and NLC groups (p < 0.05). SGR, FI, and PER were also significantly higher in the CUR-NLC group, whereas FCR did not differ significantly among treatments at this stage (p > 0.05).

**Table 3:** Growth performance and feed utilization indices of Nile tilapia receiving control diets or diets supplemented with curcumin (CUR), CUR encapsulated in nanostructured lipid carriers (CUR-NLCs), or blank nanostructured lipid carrier (NLCs) over feeding periods of 30 and 60 days.

**Time/diets**	**FW**	**WG**	**SGR**	**FI**	**FCR**	**PER**	**HSI**
30 days							
C	17.7 ± 1.1ᵃ	9.1 ± 1.1ᵃ	2.24 ± 0.19ᵃ	0.53 ± 0.03ᵃ	2.67 ± 0.35ᵃ	0.30 ± 0.04ᵃ	0.90 ± 0.14ᵃ
CUR	21.5 ± 1.2ᵃᵇ	12.9 ± 1.2ᵃ	2.90 ± 0.18ᵇᶜ	0.64 ± 0.04ᵃᵇ	1.94 ± 0.18ᵃ	0.43 ± 0.04ᵃᵇ	1.84 ± 0.22ᵇ
CUR-NLC	24.7 ± 1.5ᵇ	16.1 ± 1.5ᵇ	3.34 ± 0.20ᶜ	0.74 ± 0.04ᵇ	1.82 ± 0.20ᵃ	0.54 ± 0.05ᵇ	1.52 ± 0.17ᵃᵇ
NLC	18.3 ± 0.7ᵃ	9.7 ± 0.7ᵃ	2.44 ± 0.13ᵃᵇ	0.55 ± 0.02ᵃ	2.08 ± 0.20ᵃ	0.32 ± 0.02ᵃ	1.41 ± 0.15ᵃᵇ
60 days							
C	37.9 ± 1.0ᵃ	29.3 ± 1.0ᵃ	2.45 ± 0.04ᵃ	1.14 ± 0.03ᵃ	1.76 ± 0.06ᵃᵇ	0.97 ± 0.03ᵃ	1.57 ± 0.09ᵃ
CUR	41.1 ± 2.0ᵃ	32.5 ± 2.0ᵃ	2.55 ± 0.08ᵃ	1.23 ± 0.06ᵃ	1.96 ± 0.13ᵇ	1.08 ± 0.07ᵃ	2.06 ± 0.18ᵇ
CUR-NLC	53.0 ± 1.6ᵇ	44.4 ± 1.6ᵇ	3.01 ± 0.05ᵇ	1.59 ± 0.05ᵇ	1.64 ± 0.05ᵃ	1.48 ± 0.05ᵇ	1.79 ± 0.13ᵃᵇ
NLC	40.0 ± 1.1ᵃ	31.4 ± 1.1ᵃ	2.55 ± 0.04ᵃ	1.20 ± 0.03ᵃ	1.73 ± 0.06ᵃ	1.05 ± 0.04ᵃ	1.52 ± 0.06ᵃ

Values are presented as mean ± standard error of tank means (n = 3). Different lowercase letters within the same column and sampling time indicate significant differences among dietary treatments based on one-way analysis of variance followed by Tukey’s multiple comparison test (p < 0.05). Measured variables included FW = Final body weight, WG = Weight gain, SGR = Specific growth rate, FI = Feed intake, FCR = Feed conversion ratio, PER = Protein efficiency ratio, and HSI = Hepatosomatic index.

After 60 days of feeding, dietary differences became more pronounced. Fish receiving the CUR-NLC diet had significantly higher FW and WG than all other groups (p < 0.05), together with significantly greater FI and PER. SGR was also significantly higher in the CUR-NLC group than in the control and NLC groups (p < 0.05). In contrast, FCR remained statistically comparable among treatments, although numerically lower values were recorded in the CUR-NLC group.

Repeated-measures analysis of variance was performed to evaluate overall temporal and treatment effects. Time had a significant effect on FW, WG, FI, and PER (p < 0.05), indicating progressive changes over the experimental period. In contrast, SGR and FCR were not significantly affected by time (p > 0.05). Significant time × treatment interactions were observed for FW (p = 0.026), WG (p = 0.026), FI (p = 0.029), and PER (p = 0.023), indicating that the magnitude of temporal responses differed among dietary treatments. However, no significant interaction effects were detected for SGR or FCR (p > 0.05), indicating consistent temporal patterns across treatments for these parameters. No significant overall between-subject treatment effects were detected for FW, WG, SGR, FI, FCR, or PER in the repeated-measures model (p > 0.05). Detailed replicate tank-level datasets for growth performance, feed utilization, and HSI analyses are provided in Supplementary Table S2.

### HSI response to dietary treatments

HSI values at 30 and 60 days are presented in Table 3. At day 30, HSI differed significantly among treatments, with the CUR group showing the highest value (p < 0.05). A similar pattern was observed at day 60, when fish fed CUR showed significantly higher HSI than the control group, whereas the CUR-NLC and NLC groups showed intermediate values. Repeated-measures analysis of variance confirmed significant effects of time (p = 0.014) and treatment (p = 0.007) on HSI, whereas the interaction between time and treatment was not significant (p = 0.310), indicating a consistent temporal trend across dietary treatments.

### Length–weight relationship and growth pattern

Growth pattern analysis after 60 days of feeding demonstrated clear dietary effects on the length-weight relationship of Nile tilapia (Figures 3A–D). Estimation of the allometric growth coefficient revealed the highest b value in fish receiving the CUR-NLC diet (b = 3.278), indicating enhanced positive allometric growth. Lower b values were recorded in fish fed the CUR diet (3.045), followed by the control (2.684) and NLC (2.642) groups.

Regression analysis demonstrated strong relationships between body length and weight across all experimental diets. The CUR-NLC treatment showed the strongest linear relationship, with a correlation coefficient of 0.972 and a coefficient of determination of 0.945. Slightly lower but still strong associations were observed in the CUR group (r = 0.964; R² = 0.930), whereas the control (r = 0.951; R² = 0.905) and NLC (r = 0.947; R² = 0.897) groups showed comparatively weaker relationships. All fitted regression models were highly statistically significant (p < 0.001).

Assessment of Kn condition further supported the dietary effects on growth performance. Kn was highest in fish fed the CUR diet (1.214), followed by fish receiving CUR-NLC supplementation (1.067). In contrast, fish in the control (0.982) and NLC (0.974) groups showed lower Kn values, indicating comparatively reduced condition status. Individual fish length and body weight measurements used for regression and condition factor analyses are provided in Supplementary Table S3.

### Post-challenge survival following *S. agalactiae* infection

**Figure 4:**
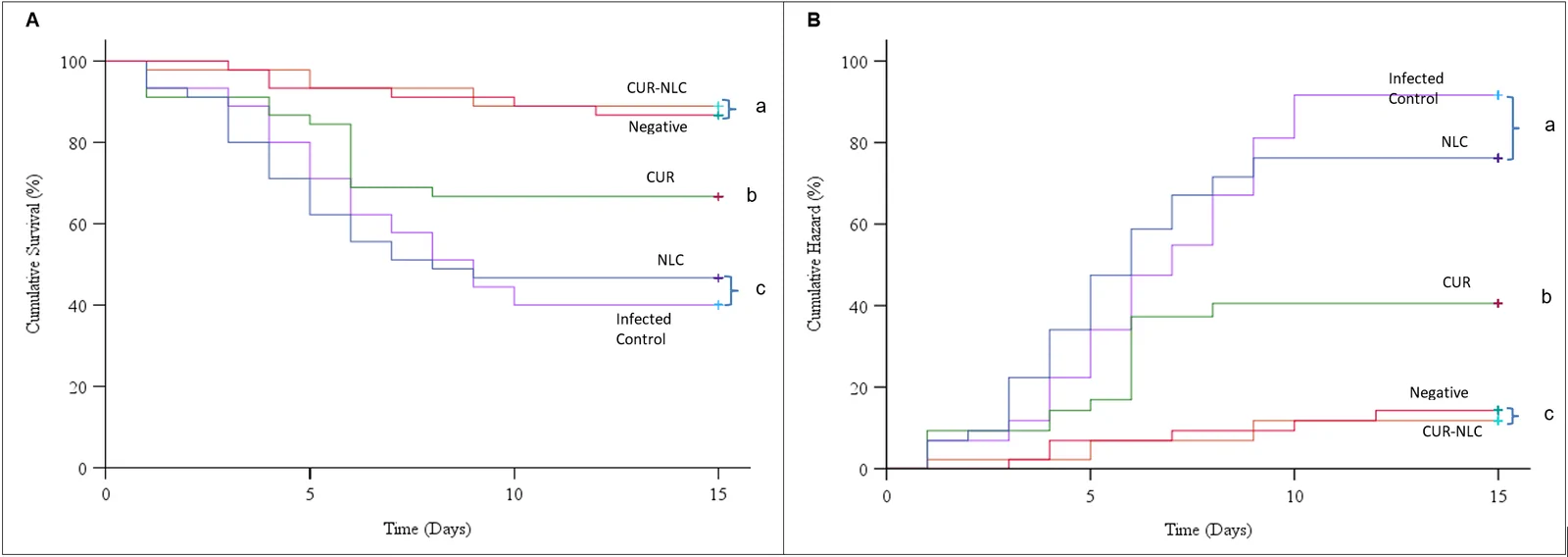
Disease resistance responses of Nile tilapia following experimental challenge with *Streptococcus agalactiae* ENC06. (A) Kaplan-Meier survival curves showing cumulative survival among dietary treatment groups during the 15-day post-challenge period. (B) Cumulative hazard (%) plots derived from Cox proportional hazards regression analysis illustrating relative mortality risk across treatments over time. Different lowercase superscripts indicate statistically significant differences among treatments (p < 0.05).

Kaplan-Meier survival curves following *S. agalactiae* ENC06 challenge are presented in Figure 4A. Survival differed significantly among treatment groups according to the log-rank (Mantel-Cox) test (χ²(4) = 40.797, p < 0.01).

Corresponding cumulative mortality, survival, and RPS values are summarized in Table 4. The infected control group showed the highest mortality (62.2 ± 5.9%) and the lowest survival (37.8 ± 5.9%), which differed significantly from the CUR-NLC and negative control groups (p < 0.05). Fish fed the CUR-NLC diet showed the lowest mortality (11.1 ± 2.2%) and highest survival (88.9 ± 2.2%), with an RPS of 82.4 ± 2.8%. Similarly, the negative control group showed low mortality (13.3 ± 3.8%) and high survival (86.7 ± 3.8%), corresponding to an RPS of 77.5 ± 7.1%. Fish fed free CUR showed intermediate responses, with mortality of 33.3 ± 3.8% and survival of 66.7 ± 3.8%, resulting in an RPS of 44.3 ± 11.1%. In contrast, the blank NLC group showed mortality (55.6 ± 4.4%) and survival (44.4 ± 4.4%) values that were not statistically different from those of the infected control group (p > 0.05), with a low RPS of 9.3 ± 11.0%. Daily cumulative survival records for each replicate tank during the post-challenge period are presented in Supplementary Table S4.

### Survival risk assessment based on Cox proportional hazards modeling

The Cox proportional hazards model indicated that survival outcomes differed significantly among dietary treatments following bacterial challenge (χ² = 38.960, df = 4, p < 0.001; Figure 4B). The cumulative hazard plot further showed that fish receiving CUR-NLC supplementation maintained the lowest relative mortality risk throughout the post-challenge period compared with all infected treatment groups.

Infected ControlCURCUR-NLCNLCNegative ControlcabAInfected ControlNLCaCURCUR-NLCNegative ControlcbBInfected ControlCURCUR-NLCNLCNegative ControlcabAInfected ControlNLCaCURCUR-NLCNegative ControlcbBInfected ControlCURCUR-NLCNLCNegative Controlcab**A**Infected ControlNLCaCURCUR-NLCNegative Controlcb**B**Infected ControlCURCUR-NLCNLCNegative Controlcab**A**Infected ControlNLCaCURCUR-NLCNegative Controlcb**B**

**Table 4:** Cumulative mortality and relative percent survival (RPS) of Nile tilapia after a 60-day feeding period with control, curcumin (CUR), CUR encapsulated in nanostructured lipid carriers (CUR-NLCs), or blank nanostructured lipid carrier (NLCs), followed by experimental infection with Streptococcus agalactiae ENC06.

**Treatments**	**Mortality (%)**	**Survival (%)**	**RPS (%)**
Infected control	62.2 ± 5.9ᵃ	37.8 ± 5.9ᵃ	0.0
Negative control	13.3 ± 3.8ᶜ	86.7 ± 3.8ᶜ	77.5 ± 7.1ᶜᵈ
CUR	33.3 ± 3.8ᵇ	66.7 ± 3.8ᵇ	44.3 ± 11.1ᵇ
CUR-NLC	11.1 ± 2.2ᶜ	88.9 ± 2.2ᶜ	82.4 ± 2.8ᶜ
NLC	55.6 ± 4.4ᵃ	44.4 ± 4.4ᵃ	9.3 ± 11.0ᵃ

Data are presented as mean values with corresponding standard error calculated from three independent replicates. Within each column, values marked with different lowercase superscript letters differ significantly among dietary treatments.

When mortality risk was evaluated relative to the infected control group, fish receiving CUR showed a significantly reduced likelihood of death, corresponding to an estimated hazard reduction of 50% (Exp(B) = 0.500; 95% confidence interval: 0.266-0.940; p = 0.031). A stronger protective effect was observed with the CUR-NLC treatment, with a markedly lower mortality risk (Exp(B) = 0.144; 95% confidence interval: 0.055-0.374; p < 0.001). In contrast, administration of blank NLCs did not alter survival probability compared with the infected control group (Exp(B) = 0.958; p = 0.878). As expected, fish in the uninfected control group showed substantially reduced mortality risk throughout the observation period (Exp(B) = 0.174; 95% confidence interval: 0.072-0.422; p < 0.001).

## DISCUSSION

This study demonstrated that CUR delivered through an NLC system produced compound-specific functional outcomes in Nile tilapia, including improved growth trajectory, feed utilization, metabolic indices such as HSI and growth allometry, and enhanced survival following *S. agalactiae* challenge. Although lipid-based nanocarriers and other nano-CUR systems have been explored for phytogenic delivery in aquatic species, CUR is a polyphenolic compound with distinct physicochemical limitations and biological targets. Therefore, sustained dietary delivery of CUR may influence oxidative balance, liver metabolism, and immune responsiveness in ways that cannot be directly extrapolated from other phytobiotic–nanocarrier studies.

### Physicochemical characteristics and implications for CUR delivery

CUR-NLCs exhibited favorable physicochemical properties suitable for oral delivery. The increase in particle diameter from 177.0 ± 3.2 nm in blank NLCs to 215.6 ± 2.6 nm in CUR-NLCs supports successful incorporation of CUR into the lipid matrix, consistent with NLC loading behavior reported for hydrophobic bioactive compounds [56, 57]. The reduced polydispersity index of CUR-NLCs (0.145 ± 0.003) compared with blank NLCs (0.269 ± 0.034) indicates a more uniform dispersion [58, 59], which is advantageous for consistent gastrointestinal exposure and uptake. Both formulations exhibited moderately negative zeta potentials (CUR-NLCs: −17.5 ± 0.78 mV; blank NLCs: −23.12 ± 0.9 mV), suggesting adequate colloidal stability through electrostatic repulsion that limits aggregation [60–62]. The high encapsulation efficiency (98.79 ± 1.51%) further confirms effective CUR entrapment [30, 63, 64]. Transmission electron microscopy imaging supported these findings by showing predominantly spherical, uniformly distributed particles of approximately 200 nm in diameter, consistent with dynamic light scattering results and indicating the structural integrity of the nanocarrier system [40, 41, 43, 65, 66].

The *in vitro* release profile supports a key delivery advantage of CUR-NLCs. CUR-NLCs exhibited a controlled and sustained-release pattern, whereas free CUR showed a rapid initial burst release followed by a slower phase. For polyphenols such as CUR, for which stability and bioavailability are major constraints, controlled-release may enable prolonged physiological exposure and improved functional effects during chronic feeding [67–69]. Despite these favorable characteristics, long-term storage stability and CUR degradation under different conditions, including temperature, pH, and feed incorporation, were not evaluated in this study. These aspects are important for practical application and should be addressed in future studies.

### Molecular interactions supporting the stabilization of CUR

Fourier-transform infrared spectroscopy results further supported effective incorporation of CUR into the lipid matrix. Shifts in the O–H stretching band at approximately 3508 cm⁻¹ suggest hydrogen bonding between CUR and lipid components, which can enhance stabilization and dispersion [70, 71]. Shifts in the C=O stretching region at approximately 1736 cm⁻¹ indicate interactions with ester linkages in the lipid matrix [72, 73]. Broadening and reduced intensity of C=C stretching at 1600–1625 cm⁻¹ and aromatic ring vibrations, together with changes in C–O stretching and C–H bending peaks at 1300–1400 cm⁻¹, reflect a modified microenvironment and strong compound–carrier interactions [74, 75]. These molecular interactions are consistent with improved stability and solubility, which are critical limitations for CUR use in aquafeeds [63, 67–69].

### Antibacterial performance of CUR-NLCs

CUR-NLCs produced larger IZ values (14–15 mm) than free CUR (6 mm), indicating improved diffusion and localized antibacterial activity on solid media. This finding is consistent with reports that nanoencapsulation improves dispersibility and contact efficiency [76, 77]. The antibacterial activity of CUR has been associated with mechanisms such as membrane disruption and interference with virulence-related processes [78]. However, MIC and MBC values remained unchanged between free CUR and CUR-NLCs, suggesting that nanoencapsulation did not fundamentally shift bactericidal thresholds under broth conditions. This pattern is biologically plausible because sustained-release systems may enhance localized exposure and prolonged interaction without necessarily achieving the rapid high concentrations required in endpoint MIC and MBC assays [79–81]. Therefore, the antibacterial findings are best interpreted as improved delivery and dispersion effects rather than altered intrinsic potency.

### Growth and feed utilization benefits

The feeding trial demonstrated that CUR-NLCs produced early improvements at 30 days and sustained improvements at 60 days in growth performance and feed utilization compared with free CUR, blank NLCs, and control diets. These effects address a key limitation of CUR, namely poor solubility and low oral bioavailability, and support the interpretation that NLC delivery improved functional exposure to CUR during chronic feeding [82]. Mechanistically, NLCs may protect CUR from degradation in the gastrointestinal tract and facilitate uptake by enhancing interactions with intestinal surfaces, thereby improving absorption efficiency [69, 83]. Sustained availability may also support the antioxidant, physiological, and health-promoting effects of CUR, reducing physiological stress and enabling more efficient nutrient partitioning toward growth [84].

The improvement in FCR in the CUR-NLC group, particularly by day 60, indicates enhanced feed efficiency, potentially linked to improved nutrient utilization and metabolic efficiency under sustained CUR exposure [34, 47, 85]. The increased PER further suggests improved protein utilization, potentially reflecting reduced oxidative catabolism and improved nitrogen retention under the antioxidant and immune-supportive effects of CUR [47, 85, 86]. Gut microbiota composition was not evaluated in the present study; therefore, potential microbiome-mediated contributions to improved feed efficiency and health performance associated with CUR-NLC supplementation remain unclear. Although free CUR improved performance relative to the control, its effects were consistently lower than those of CUR-NLCs, highlighting the importance of delivery constraints in determining *in vivo* efficacy. Because identical nominal CUR concentrations were used in the free CUR and CUR-NLC diets, the improved biological responses observed in the CUR-NLC group are more likely attributable to enhanced delivery efficiency and sustained bioavailability rather than differences in dietary inclusion level alone. The absence of visible feed rejection and apparent CUR leaching during feeding further supports the practical suitability of CUR-NLC-coated pellets for dietary application in Nile tilapia culture systems.

### Metabolic indices and growth allometry

HSI outcomes provided additional evidence of functional metabolic effects. By 60 days, both CUR and CUR-NLCs increased HSI compared with the control, with CUR showing the highest HSI. These findings may reflect metabolic responses associated with prolonged dietary CUR exposure [87, 88], although direct evidence supporting a hepatoprotective effect was not evaluated in the present study. Histopathological evaluation of hepatic or intestinal tissues and serum biochemical markers, such as alanine aminotransferase and aspartate aminotransferase, were not assessed. Therefore, interpretations regarding hepatoprotective effects remain preliminary and require further investigation.

The positive allometric growth observed in the CUR-NLC group (b = 3.278) indicates proportionally greater WG relative to length, suggesting favorable nutrient allocation and tissue deposition under sustained CUR delivery [30, 89, 90]. In contrast, the control and NLC groups exhibited negative allometry, supporting the conclusion that CUR, rather than the carrier alone, contributed to the improved growth pattern [50]. Although free CUR produced the highest Kn value, CUR-NLCs enhanced growth dynamics the most, indicating that the effects of CUR on body condition and weight deposition may not be identical and may vary with delivery form and exposure kinetics [30, 90].

### Disease resilience and survival modeling

The most biologically and commercially relevant outcome was improved disease resilience following *S. agalactiae* challenge. CUR-NLC-fed fish exhibited the greatest survival and lowest mortality, with an RPS of 82.4 ± 2.8%. Cox regression further indicated an approximately 86% reduction in mortality risk relative to infected controls, whereas free CUR reduced the risk by approximately 50%. The minimal effect of blank NLCs confirms that the protective outcome was primarily mediated by CUR rather than the carrier matrix.

Given that MIC and MBC values were unchanged, the improved survival response observed in the CUR-NLC group may be associated with enhanced CUR bioavailability and sustained physiological exposure, which could contribute to improved host resilience during infection rather than direct bactericidal activity alone [30, 34, 91]. In practical terms, these findings support CUR-NLCs as a phytobiotic-based approach to reducing antibiotic dependence in streptococcosis-prone production systems, particularly in regions where *S. agalactiae* is endemic and recurrent.

Although improved post-challenge survival may suggest enhanced physiological resilience, hematological parameters, antioxidant biomarkers, innate immune responses, tissue bacterial burden, and immune-related molecular responses were not evaluated in the present study. Therefore, the precise mechanisms underlying enhanced disease resistance, including potential effects on systemic bacterial colonization, immunomodulation, and antioxidant-related pathways, remain unclear and require further investigation. Furthermore, although no overt adverse effects were observed in fish fed the blank NLC diet, a comprehensive toxicological evaluation of the nanocarrier system was beyond the scope of this study. Future investigations should therefore include detailed immunophysiological assessments together with long-term biosafety evaluations, including histopathological analysis, nanoparticle accumulation, and environmental fate associated with prolonged dietary exposure.

### Translational relevance and future application

From a translational perspective, the CUR-NLC formulation used in this study may be adaptable for aquaculture applications because it uses commonly available lipid excipients and a relatively simple preparation process. Although nanoencapsulation may increase production costs compared with free CUR, improved bioavailability, feed efficiency, and post-challenge survival could potentially offset these costs. However, the economic feasibility and scalability of CUR-NLC incorporation into industrial feed manufacturing systems require further investigation through techno-economic analyses and farm-scale validation studies.

In addition, although CUR and the lipid excipients used in the present NLC formulation are generally regarded as biodegradable and biocompatible, the current study did not directly assess tissue residue accumulation, degradation behavior, or potential release of formulation components into surrounding aquatic systems. Because lipid-based nanocarriers may influence the stability, delivery efficiency, and biological distribution of encapsulated compounds, further studies evaluating residue kinetics, biodegradation dynamics, and environmental safety under commercial aquaculture conditions are warranted to support the sustainable application of CUR-NLCs.

## CONCLUSION

Dietary supplementation with CUR-NLCs improved growth performance, feed utilization efficiency, and resistance to *S. agalactiae* infection in Nile tilapia. CUR-NLCs showed favorable physicochemical characteristics, including nanoscale particle size, uniform dispersion, high encapsulation efficiency, and sustained *in vitro* release. Fish receiving CUR-NLCs had higher FW, WG, SGR, FI, and PER, and demonstrated positive allometric growth after 60 days of feeding. Following bacterial challenge, CUR-NLC-fed fish showed the lowest mortality (11.1 ± 2.2%), highest survival (88.9 ± 2.2%), and greatest RPS (82.4 ± 2.8%), with Cox regression indicating an approximately 86% reduction in mortality risk compared with infected controls.

These findings suggest that NLC-based delivery enhances the biological efficacy of CUR by improving its dispersion, sustained availability, and functional performance in aquafeeds. In practice, CUR-NLCs may serve as a promising phytobiotic-based nutritional strategy to improve productivity and disease resilience in streptococcosis-prone tilapia culture systems, potentially reducing reliance on conventional antimicrobials.

A major strength of this study is the integrated evaluation of CUR-NLCs, combining physicochemical characterization, antibacterial testing, growth assessment, feed utilization indices, growth allometry, and post-challenge survival modeling. However, the study was limited by the absence of long-term storage stability testing, tissue residue evaluation, histopathology, antioxidant and immune biomarker analyses, gut microbiota profiling, and farm-scale validation. Future studies should investigate the mechanisms underlying CUR-NLC-mediated protection, including immune modulation, oxidative stress responses, bacterial tissue burden, nanoparticle biodistribution, environmental fate, and economic feasibility under commercial aquaculture conditions.

Overall, CUR-NLCs represent a sustainable and biologically functional feed additive with potential application in Nile tilapia health management and production improvement.

## DATA AVAILABILITY

The supplementary data can be made available from the corresponding author upon request. 

## GENERATIVE AI DECLARATION

 The authors declare that no generative artificial intelligence (AI) or AI-assisted technologies were used in the writing, analysis, or preparation of this manuscript.

## AUTHORS’ CONTRIBUTIONS

WK, MTK, and NP: Conceptualization. WK, MTK, SK, JY, GS, and NP: Methodology. WK and MTK: Software. WK, MTK, SK, JY, and NP: Validation. SK, JY, GS, SVM, and NP: Formal analysis. WK, MTK, and NP: Investigation. WK, MTK, SK, JY, GS, KDT, SVM, and SJ: Data curation. WK: Writing—original draft preparation. WK, MTK, SK, JY, GS, KDT, SVM, SJ, and NP: Writing—review and editing. MTK: Visualization. MTK and NP: Supervision. NP: Project administration. All authors have read and approved the final manuscript.
